# Illusory palinopsias induced by *in vitro* fertilization treatment: a case report

**DOI:** 10.1186/s13256-019-2115-7

**Published:** 2019-05-30

**Authors:** Ramesh Venkatesh, Naresh Kumar Yadav, Shivani Sinha

**Affiliations:** Department of Retina and Vitreous, Narayana Nethralaya, #121/C, 1st R block, Chord Road, Rajaji Nagar, Bengaluru, 560010 India

**Keywords:** Side effects, Estrogen, Follicle-stimulating hormone, Pregnancy, *In vitro* fertilization, Case report

## Abstract

**Background:**

In the current era, *in vitro* fertilization, a type of assisted reproductive technology, has been commonly used for infertility management and gestational surrogacy. The techniques that are routinely used in *in vitro* fertilization include ovarian hyperstimulation to generate multiple eggs, preparation of the ova and sperm, and culture and selection of resultant embryos before transfer into a uterus. These steps increase the chances of successful pregnancy following *in vitro* fertilization treatment many-fold, especially in young women. Complications reported with *in vitro* fertilization treatment include multiple gestations, ovarian hyperstimulation syndrome, and birth defects while ocular side effects reported include retinal detachment and progression of keratoconus. We report a case of visual illusory palinopsia following *in vitro* fertilization treatment in a patient with unexplained infertility.

**Case presentation:**

A 31-year-old Asian woman was administered *in vitro* fertilization treatment for her unexplained infertility. She complained of visually disturbing flashes in her peripheral vision during her pregnancy. She described these flashes as occurring usually in the morning hours or while walking, coming in sets of three to four, occurring five–six times a day and lasting for less than 5–10 minutes. Her flashes were not accompanied by other ocular symptoms such as pain, redness, photophobia, or decrease in vision. Her ocular examination was normal. Neuroimaging with magnetic resonance imaging revealed no pathology. A diagnosis of visual illusory palinopsia secondary to *in vitro* fertilization treatment was made.

**Conclusion:**

Disturbing visual palinopsia and afterimages can occur following *in vitro* fertilization treatment for infertility due to increased estrogen levels. This rare ocular side effect caused by *in vitro* fertilization treatment is not reported in the literature to the best of our knowledge. Gynecologists and/or infertility experts should educate their patients regarding these possible ocular symptoms. Even ophthalmologists should be aware of this rare cause for visual palinopsia.

## Introduction

*In vitro* fertilization (IVF) is a type of assisted reproductive technology (ART) used for infertility treatment and gestational surrogacy, in which a fertilized egg is implanted into the same or another woman’s uterus [1, 2]. Recent modifications and improvements in IVF have expanded its indications for use, which has become a common procedure in ART [3]. Problems with IVF treatment include multiple gestations, ovarian hyperstimulation syndrome, and birth defects while ocular side effects like retinal detachment and keratoconus have been reported [4, 5]. We report a case of visual illusory palinopsia following IVF treatment in a patient with unexplained infertility and explain the possible pathomechanism.

## Case presentation

A 31-year-old Asian woman diagnosed as having unexplained infertility decided to undergo IVF treatment to achieve a successful pregnancy. She had no past systemic illness like diabetes mellitus or hypertension. She was started on birth control pills, Ovral L tablets (ethinyl estradiol 0.03 mg + levonorgestrel 0.15 mg), to prevent pregnancy before commencing IVF treatment. Daily injections of Gonal-f® (follitropin alfa injection) 225 IU were given during which time the stimulation was monitored using a combination of vaginal ultrasound and blood estrogen level every 2–3 days. The inability of blood estrogen levels to rise adequately prompted the physician to add 450 IU injectable Menopur®, which comprises 75 IU follicle- stimulating hormone (FSH) + 75 IU luteinizing hormone (LH), for multiple egg creation. Injectable Cetrotide® (cetrorelix acetate for injection) 0.25 mg subcutaneously was given for 5 days to prevent premature ovulation. Injectable Ovitrelle® (choriogonadotropin alfa) 250 μg/0.5 ml was given subcutaneously to prepare the largest mature follicles for ovulation. The egg was retrieved, fertilization was achieved, and embryo was transferred to our patient’s uterus for implantation. After embryo transfer, she was started on Endofert tablets (estradiol valerate) 2 mg daily for 2 months along with Susten tablets (progesterone) 200 mg twice daily supplements for the entire length of pregnancy. She had no high blood pressure or blood sugar during her pregnancy. She had a twin delivery. Currently, she is in her third month of post-partum period. She complained of seeing disturbing flashes in peripheral vision beginning in her third trimester. She described these flashes as usually occurring in the morning hours or while walking, coming in sets of three to four, occurring five–six times a day and lasting for less than 5–10 minutes. She says that her symptoms occur even now; however, with reduced frequency. Her flashes were not accompanied by other ocular symptoms such as pain, redness, photophobia, or decrease in vision. She gave no past or family history of migraine. She visited many retina specialists with complaints of persistence of symptoms. Her ocular examination was normal. A physician’s and a neurologist’s opinion were sought to rule out migraine. Plain magnetic resonance imaging (MRI) of her brain was normal. A diagnosis of IVF treatment-induced visual illusory palinopsia was suspected. She was counselled and reassured regarding her symptoms.

## Discussion

We describe an interesting case of illusory palinopsia following IVF treatment in a woman diagnosed as having unexplained infertility.

Estrogen plays a vital role in follicular development, ovulation, and pregnancy if conception occurs. Treatment with ART includes the use of ovulation- induction drugs like clomiphene citrate or treatment with FSH analogs or gonadotropin-releasing hormone antagonists and human chorionic gonadotropin. Palinopsias are visual disturbances characterized by persistent recurrence of a visual image after the stimulus has been withdrawn. Palinopsias are grouped into two categories: illusory palinopsias and hallucinatory palinopsias [6]. Illusory palinopsias are caused by migraines, head trauma, prescription drugs, or hallucinogen-persisting perception disorder. The afterimages in illusory palinopsias are affected by ambient light and motion and are unformed, indistinct, or low resolution similar to that described by our patient. Hallucinatory palinopsias are due to posterior cortical lesions. An MRI of her brain did not show any neurological lesions. Migraine was considered one of the differential diagnoses in this case. However, our patient had no family or past history of migraine and her symptoms were not followed by migraine-like headache. A physician’s and a neurologist’s opinion were also sought to rule out migraine which was absent. Pre-eclampsia or eclampsia-induced visual symptoms were ruled out as our patient did not have high blood pressure or pedal edema during her pregnancy. Hence, her symptoms could be secondary to the IVF treatment. Illusory palinopsias are caused by diffuse neuronal pathology such as global alterations in neurotransmitter receptors. Yilmaz *et al.* [7] have shown different patterns of visually evoked potential latencies during different phases of the menstrual cycle. The latencies are reduced during the follicular and ovulatory phases while they are increased during the ovulatory phase of the menstrual cycle. Estrogen inhibits ɤ-aminobutyric acid synthesis, an important inhibitory neurotransmitter in the cerebral and visual cortexes and is involved in the genesis of visually evoked potentials. The inhibition of ɤ-aminobutyric acid reportedly increases the excitatory effect on the striate cortex [8]. Thus, estrogen can directly or indirectly stimulate the visual cortex, thus triggering the development of visual hallucinations. A functional MRI (fMRI) to check for the cortical activation areas during the symptoms could have been useful. However, in our case the symptoms lasted for < 10 minutes and therefore it was practically not possible for her to undergo fMRI. Visual hallucinations following treatment with ovulation induction drugs like clomiphene citrate due to a similar pathomechanism has also been reported [9, 10]. Keratoconus progression due to increase in estrogen levels following IVF treatment has been reported by Yuksel *et al.* [5]. Ratson *et al*. [4] reported a higher risk of developing retinal detachment following IVF treatment. In our case, we believe that the visual symptoms described were secondary to increased estrogen levels due to IVF treatment.

## Conclusion

Visual palinopsias and afterimages can occur following IVF treatment due to increased estrogen levels. Gynecologists and/or infertility experts should educate their patients regarding these possible ocular symptoms. Even ophthalmologists should be aware of this unusual cause for visual disturbance.

## Abbreviations

ART: Assisted reproductive technology
fMRI: Functional magnetic resonance imaging
FSH: Follicle-stimulating hormone
IVF: *In vitro* fertilization
MRI: Magnetic resonance imaging
